# Biocompatible Core–Shell-Structured Si-Based NiO Nanoflowers and Their Anticancer Activity

**DOI:** 10.3390/pharmaceutics14020268

**Published:** 2022-01-23

**Authors:** Kihak Gwon, Jong-Deok Park, Seonhwa Lee, Jong-Sung Yu, Do Nam Lee

**Affiliations:** 1 Ingenium College of Liberal Arts (Chemistry), Kwangwoon University, Seoul 01897, Korea; khgwon@kw.ac.kr (K.G.); seonhwalee@kw.ac.kr (S.L.); 2 Department of Physiology and Biomedical Engineering, Mayo Clinic, Rochester, MN 55902, USA; 3 Department of Energy Science and Engineering, Daegu Gyeongbuk Institute of Science and Technology (DGIST), Daegu 42988, Korea; jd3388@dgist.ac.kr

**Keywords:** core–shell, Ni composite, nanoflowers, biocompatibility, anticancer activity

## Abstract

Compared to most of nano-sized particles, core–shell-structured nanoflowers have received great attention as bioactive materials because of their high surface area with the flower-like structures. In this study, core–shell-structured Si-based NiO nanoflowers, Si@NiO, were prepared by a modified chemical bath deposition method followed by thermal reduction. The crystal morphology and basic structure of the composites were characterized by powder X-ray diffraction (PXRD), Fourier-transform infrared spectroscopy (FT-IR), scanning electron microscopy (SEM), transmission electron microscopy (TEM), specific surface area (BET) and porosity analysis (BJT), and inductively coupled plasma optical emission spectrometry (ICP-OES). The electrochemical properties of the Si@NiO nanoflowers were examined through the redox reaction of ascorbic acid with the metal ions present on the surface of the core–shell nanoflowers. This reaction favored the formation of reactive oxygen species. The Si@NiO nanoflowers showed excellent anticancer activity and low cytotoxicity toward the human breast cancer cell line (MCF-7) and mouse embryonic fibroblasts (MEFs), respectively, demonstrating that the anticancer activities of the Si@NiO nanoflowers were primarily derived from the oxidative capacity of the metal ions on the surface, rather than from the released metal ions. Thus, this proves that Si-based NiO nanoflowers can act as a promising candidate for therapeutic applications.

## 1. Introduction

Cancer is a disease that is associated with abnormal cell growth and can spread to other parts of the body. Globally, cancer-associated deaths are only second in number after deaths related to cardiovascular diseases and are considered the most critical global health issue. Conventional drugs and diagnostics for cancer therapies suffer from poor absorption from target sites, require repeated dosing, and are associated with high dose dumping. Consequently, a new and improved technology is required for effective therapeutic drug loading and theranostics to ensure suitable diagnostics in pre-stage cancer [1,2,3]. At present, healthcare providers and researchers are working in communion to develop effective cancer therapies [4]. Nanotechnology-based drug systems and theranostics are commonly based on nanoparticles (NPs) that are 10–1000 nm in size, and various nanotechnologies based on liposomes, micelles, dendrimers, CNTs, quantum dots, and other composites have been developed to enhance the efficacy of drugs, improving the solubility of hydrophobic drugs and adequately controlling the drug release [5,6,7,8,9,10,11,12,13]. NPs have unique and favorable properties that are different from those of bulk materials in terms of drug toxicity and bio-distribution. Due to its outstanding features, nanotechnology has emerged as a new solution for cancer therapy. However, the small sizes of the NPs may occasionally have adverse effects because of nonspecific accumulation in the organs, including in the liver, spleen, and lung, resulting in cytotoxicity [14,15]. This has hindered the extensive application of various NPs in cancer therapy, although a variety of nano drugs, including Dosil^®^, Eligard^®^, Abraxanes^®^, Genexol PM^®^, and Onivyde^®^, have been approved by the FDA [16,17,18,19,20]. The suitability of noble metal NPs, including Ag, Au, and Pt NPs, as biomaterials has been intensively studied for therapeutic treatments [21,22,23,24,25]. On the other hand, metal oxides such as CuO, ZnO, TiO_2_, and NiO have been introduced as new alternatives that exhibit good antimicrobial activities [26,27,28,29,30]. In particular, ZnO NPs are widely used in cancer therapy, as they are toxic to cancer cells; however, they show high cytotoxicity at high doses. The methylation of histidine resulted in increased anticancer activity at low doses for bladder cancer T-24 cells, leading to the discovery of a novel epigenetic mechanism with low toxicity at low concentrations of ZnO [31,32,33]. Furthermore, metal oxide NPs containing Fe, Co, and Ni are of great interest due to their unique optical, electronic, and physiochemical properties that can be harnessed in various fields such as photo catalysis, drug delivery, catalysis, magnetic resonance imaging, and cancer therapy [34,35,36,37,38,39,40,41]. In nanotechnology, particle size and morphology are crucial for determining the potency for medicinal applications [42,43,44,45]. NiO NPs exhibit excellent cytotoxic effects due to their unique properties, such as metal ion release, high surface area to volume ratio, enhanced adsorbing ability, and lower toxicity, compared to other metal oxides exhibiting anticancer activity [46]. Compared to nanostructures such as nanospheres or nanorods, nanoflower-like structures with different petals provide high surface areas [43,47,48]. However, a survey of the literature indicates that only a few studies have investigated metal oxide-based nanoflowers for anticancer reagents.

In this study, we explored new flower-like NiO NPs that exhibited high anticancer activity and low cytotoxicity. Core–shell-structured Si-based NiO (Si@NiO) nanoflowers were prepared by a modified chemical deposition method followed by thermal reduction. Their physical and electrochemical properties were investigated using various microscopic, spectroscopic, and electrochemical techniques. The therapeutic potential of the Si@NiO nanoflowers was investigated by testing the viability of the human breast cancer cell line MCF-7 in the presence of Si@NiO and the cytotoxicity of Si@NiO toward mouse embryonic fibroblasts (MEFs). The potential therapeutic properties of Si@NiO and its anticancer mechanism are presented.

## 2. Materials and Methods

### 2.1. Synthesis of Si@NiO Nanoflowers

Si@NiO nanoflowers were synthesized by chemical bath deposition (CBD) and subsequent reductive heating, as reported previously [48]. To synthesize the Si@NiOOH nanoflowers, plasma-synthesized Si NPs (20 mg, particle size: 50–100 nm, 99% pure) purchased from Sigma-Aldrich (St. Louis, MO, USA) were dispersed in a beaker containing a mixture of water (4 mL) and ethanol (2 mL) and were sonicated for 2 h (Solution 1). Ni(CH_3_COO)_2_·4H_2_O (0.25 g), and potassium peroxodisulfate (0.05 g) was dissolved in a beaker containing water (4 mL) and ethanol (2 mL) (Solution 2). Solution 1 was mixed with Solution 2 under magnetic stirring at 200 rpm (Solution 3). NH_4_OH (28–37%, 0.2 mL) was diluted in deionized water (10 mL) and added dropwise (one drop per second) to Solution 3 under stirring. The yellowish solution slowly turned dark after several minutes. The reaction continued for 30 min following the addition of NH_4_OH, and the precipitate was washed with a large amount of deionized water to remove the adsorbed ions (SO_4_^2−^, K^+^, Ni^2+^, CH_3_COO^−^, etc.). The brown Si@NiOOH nanoflowers that were obtained were dried in an oven at 60 °C for 6 h. The dried Si@NiOOH nanoflowers were transformed to NiO-coated Si (Si@NiO) by heating the former under Ar flow in a tube furnace under increasing temperatures up to 350 °C at a heating rate of 2 °C min^−1^. The sample was heated using a temperature control program and then naturally cooled to ambient temperature [48].

### 2.2. Characterization of Si@NiO Nanoflowers

The morphologies of the Si NPs and Si@NiO nanoflowers were characterized by scanning electron microscopy (SEM; Hitachi SU8230, Hitachi High-Tec, Tokyo, Japan) at an acceleration voltage of 10 kV and transmission electron microscopy (TEM; Tecnai G2 F20 TWIN TMP, FEI, Hillsboro, OR, USA) at 120 kV. Powder X-ray diffraction (PXRD) patterns of the Si NPs and Si@NiO nanoflowers were recorded using a diffractometer (SmartLab, Rigaku, Tokyo, Japan) with Cu Kα radiation and a Ni filter. The voltage and current in the X-ray tube of the diffractometer were maintained at 40 kV and 30 mA, respectively, and the patterns were acquired at a scan rate of 4°/min and at an interval of 0.02°. Fourier-transform infrared (FT-IR) spectra were recorded on a Bio-Rad FTS 135 spectrometer (Hercules, CA, USA) using KBr pellets. Thermal properties were evaluated by thermogravimetric analysis (TGA; TG 209 F3 Tarsus, NETZSCH, Selb, Germany). The specific surface areas of the samples were measured at −196 °C using the the Brunauer–Emmett–Teller (BET) method using a 3FLEX surface characterization analyzer and physisorption analyzer (Micromeritics, Atlanta, Georgia, USA). The pore size distribution was measured from the adsorption branch using the Barrett–Joyner–Halenda (BJH) method.

### 2.3. Electrochemical Properties of Si@NiO Nanoflowers

Electrochemical properties were measured at 25 °C in a traditional three-electrode system using a glassy carbon electrode (GCE) with Si@NiO sample as the working electrode, Ag/AgCl with saturated KCl as the reference electrode, and Pt wire as the counter electrode [49]. Material inks were prepared by mixing 4 mg of Si@NiO and 0.1 mL of 5% Nafion solution (Sigma-Aldrich). The inks were dropped onto a GCE. Cyclic voltammetry (CV) measurements were carried out in phosphate-buffered saline (PBS) with 0.01 M of ascorbic acid (AA) at a scan rate of 100 mV/s and voltage window of 0.0–1.0 V.

### 2.4. Metal Ion Release from Si@NiO Nanoflowers

The release of metal ions from the Si@NiO nanoflowers on tryptic soy agar plates was tested. Si@NiO (1 mg) was added to 0.9% saline solution (1 mL) and stirred for 6–48 h at 25 °C [50]. This solution was centrifuged at 25 °C and 5000 rpm for 5 min, and the supernatant was separated from the reaction tube. The metal ions that were released in the samples were identified using inductively coupled plasma optical emission spectrometry (ICP-OES; iCAP 7400 ICP-OES Duo, Thermo Fisher Scientific, Waltham, MA, USA).

### 2.5. Cell Culture

Human breast cancer cell line (MCF-7) and mouse embryonic fibroblasts (MEFs)were cultured in high-glucose Dulbecco’s modified Eagle’s medium (DMEM, Gibco Laboratories, Grand Island, NY, USA) containing 10% heat-inactivated fetal bovine serum, 100 units/mL penicillin, and 100 μg/mL streptomycin (Invitrogen, Carlsbad, CA, USA) at 37 °C in a 5% CO_2_ humidified atmosphere. The medium was changed every two days, and the cells were digested with trypsin and were re-suspended in a fresh medium before confluence.

### 2.6. In Vitro Anticancer Activity

Cell viability was quantitatively determined using the MTS (3-(4,5-dimethylthiazol-2-yl)-5-(3-carboxymethoxyphenyl)-2-(4-sulfophenyl)-2H-tetrazolium) assay, which is similar to the MTT (3-(4,5-dimethylthiazol-2-yl)-2,5-diphenyltetrazolium bromide) assay and measures the metabolic rates of cells [51,52]. MCF-7 and MEFs were seeded into a 96-well plate (2 × 10^4^ cells per well) in DMEM medium. After 12 h, the medium was replaced with 200 μL of fresh medium containing various concentrations of Si@NiO (0–200 μg/mL). After incubation for 24 h, the Si@NiO-containing media was carefully removed, and the cells were washed twice with PBS. Then, an MTS cell proliferation assay kit solution (20 µL) and fresh medium (200 µL) were added to each well. After incubation for an additional 4 h, the absorbance was measured at 490 nm using a microplate reader (Synergy H1, BioTek, Winooski, VT, USA). The number of proliferating cells was quantified using a standard curve of the cells. In addition, the cell viability was quantitatively analyzed using a live/dead assay (ThermoFisher Scientific, Waltham, MA, USA), in which the live and dead cells were identified by green and red fluorescence, respectively [51,52,53]. For this, MCF-7 and MEFs were seeded in a 48-well plate at a density of 5 × 10^4^ cells per well. After 12 h, the medium was replaced with 200 μL of fresh medium containing various concentrations of Si@NiO (0–200 μg/mL). After incubation for 24 h, the cells were rinsed with PBS and were then incubated in PBS containing 4 μM calcein-AM and 2 μM ethidium homodimer-1 (EtBr-1) for 30 min. After washing with PBS, the stained cells were observed using an inverted fluorescence microscope (IX83, Olympus, Center Valley, PA, USA). Cell viability was quantified by calculating the ratio of live cells to the total number of cells.

### 2.7. Statistical Analysis

All data are expressed as mean ± standard deviation, and experiments were performed at least in triplicate. Statistically significant differences were evaluated using the t-test, and statistical significance was as follows: (*) *p* < 0.05, (**) *p* < 0.01, and (***) *p* < 0.001.

## 3. Results and Discussion

### 3.1. Preparation of Si@NiO Nanoflowers

**Figure 1:**
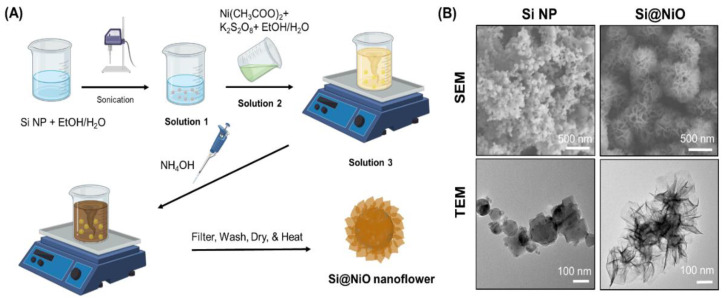
Synthesis and characterization of Si@NiO nanoflowers. (**A**) Schematic representation of the synthesis of Si@NiO nanoflowers. (**B**) SEM and TEM images of Si NPs and Si@NiO nanoflowers.

Si@NiO nanoflowers were prepared using a previously reported method [48] with slight modifications, as shown in Figure 1A. During CBD, the Si NPs were encapsulated in porous flower-like NiOOH shells (i.e., Si@NiOOH), which were subsequently reduced to a porous flower-like NiO shell to produce Si@NiO upon mild heating at 350 °C under an argon flow. The SEM and TEM images showed that the Si NPs were spherical, with an average size of 100 nm, while Si@NiO had a flower-like core–shell structure (Figure 1B), where the Si core was uniformly covered by a thin nanoflower-like NiO shell. The flower-like morphology with an open petal structure on the surface was stable and well maintained, even after further annealing. As reported previously [48], the shell layers had a uniform thickness of 10–30 nm, as observed by TEM (Figure 1B, bottom).

### 3.2. Characterization of Si@NiO Nanoflowers

**Figure 2:**
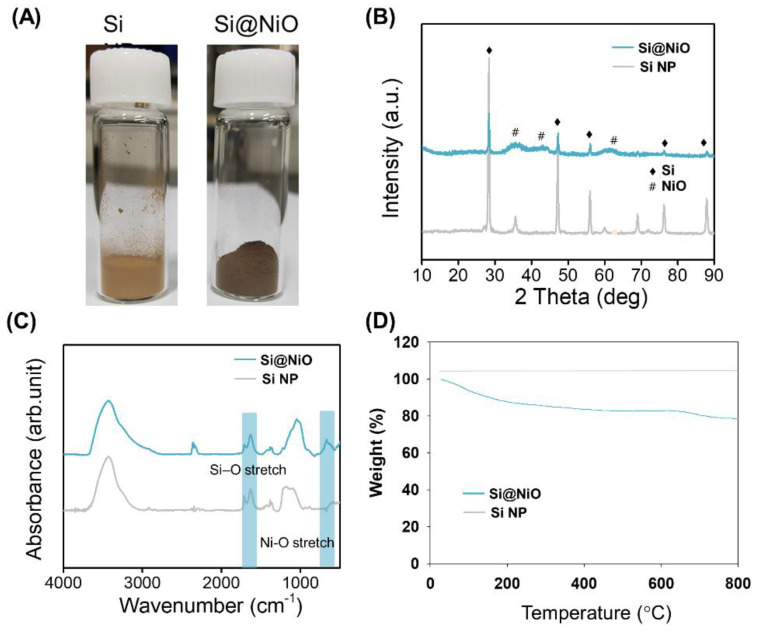
Characterization of Si@NiO nanoflowers. (**A**) Photographs of Si NP and Si@NiO. (**B**) PXRD patterns, (**C**) FT-IR spectra, and (**D**) TGA curves of Si NP and Si@NiO in air atmosphere.

As shown in Figure 2A, the bright brown color of the Si NPs changed to dark brown after the successive heat treatment of the Si substrate to form Si@NiO. The flower-like Si@NiO species was composed of a core–shell structure in which the Si NPs were wrapped by porous.

NiO sheets, as evident from the SEM and TEM images in Figure 1B. The PXRD patterns and FT-IR spectra of the Si NPs and Si@NiO were recorded. As shown in Figure 2B, high-intensity diffraction peaks were observed at 2θ values of 28.3°, 47.2°, 55.9°, 75.7°, and 87.9°, corresponding to Si (111), (220), (311), (400), and (331) lattice structure [54], respectively, and additional peaks were observed at 2θ values of 37.1°, 43.3°, and 62.6°, corresponding to the coated NiO (111), (200), and (220), respectively. (JCPDS Card No. #47-1049). The FT-IR spectrum of Si@NiO (Figure 2C) shows peaks at 685 and 1644 cm^−1^, corresponding to Ni-O stretching (NiO) and Si–O stretching (SiO_2_), respectively. This is consistent with previously reported data [55], emphasizing that Si@NiO is well synthesized from Si NP. The thermal stability of the Si NP and Si@NiO was investigated by TGA, wherein Si@NiO was found to have a high silicon content of 78.6 wt.% (Figure 2D). These observations are consistent with the findings of a previous study [48].

### 3.3. Pore Structure Characterization

**Figure 3:**
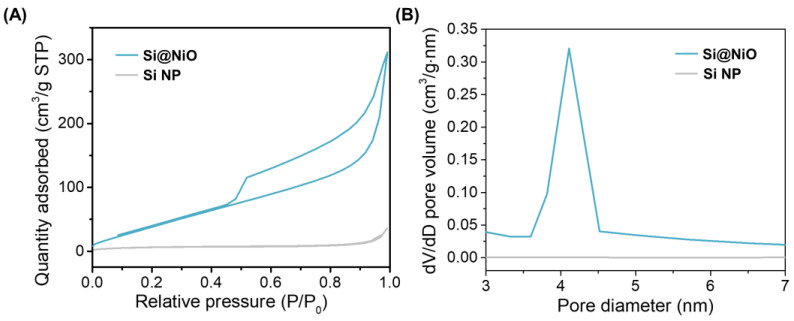
Pore structure characterization of Si@NiO nanoflowers. (**A**) N_2_ adsorption–desorption isotherms and (**B**) pore size distribution of Si NPs and Si@NiO based on the BJH method.

The pore structure and surface area of the Si NP and Si@NiO nanoflowers were evaluated from the N_2_ adsorption–desorption isotherms acquired at −196 ℃. The micropore volume (V_micro_), mesopore volume (V_meso_), and specific surface area (S_BET_) were calculated using the BET method, and the average pore size was measured from the adsorption branch using the BJH method. The N_2_ adsorption–desorption isotherms and the corresponding pore size distribution curves are shown in Figure 3A,B, respectively. The N_2_ adsorption curves for Si@NiO show a hysteresis loop from 0.5 to 1 (P/P^o^), indicating that the Si@NiO is rendered highly mesoporous after forming a shell with the NiO petal layers. On the contrary, the Si NPs showed negligible N_2_ adsorption and a low average pore size (0.56 nm), indicting their low porosity. As shown in Table 1, V_micro_, V_meso_, S_BET,_ and the average pore size significantly increased after coating the Si NPs to form Si@NiO. In particular, the larger surface area of Si@NiO, compared to those of Si NPs and other reported absorbents such as MoS_2_ and TiO_2_, can be expected to provide more favorable sites for easy attachment to cell membranes [56,57].

### 3.4. Electrochemical Properties

The CV curves of Si@NiO were obtained in PBS containing 0.1 M AA (C_6_H_8_O_6_) at a scan rate of 100 mVs^−1^ in the potential range of 0.0–1.0 V using an Ag/AgCl reference electrode. The Ni(II) center of the Si@NiO participates in the following redox reaction with AA:

Ni^2+^ + C_6_H_8_O_6_ (AA) → Ni + 2H^+^ + C_6_H_6_O_6_

**Figure 4:**
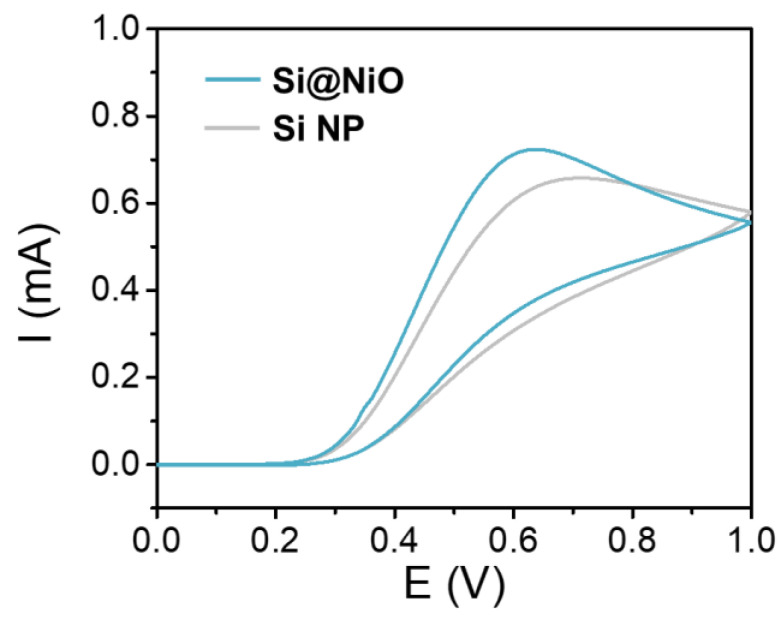
Electrochemical characterization. CV curves of Si NP and Si@NiO acquired in PBS containing 0.1 M AA; scan rate: 100 mVs^−1^.

Both Si@NiO and Si NPs were easily reduced in the redox reaction with AA, and the positive redox current was higher for Si@NiO than it was for Si NP at low potentials (Figure 4). As Si@NiO was reduced more easily by AA, it could induce reactive oxygen species (ROS) formation more easily than the Si NPs could.

### 3.5. Ion Release Test

**Figure 5:**
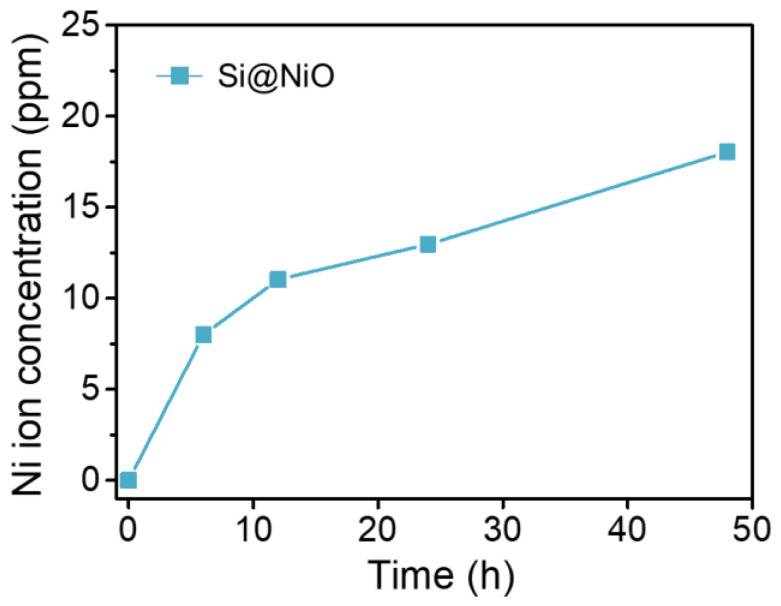
Metal ion release from Si@NiO nanoflowers. Concentration of Ni ions released from 1 mg/mL of Si@NiO in 0.9% saline solution.

The amount of Ni ions released from the Si@NiO nanoflowers was measured using ICP-OES. Si@NiO degradation was tested at a concentration of 1 mg/mL in 0.9% saline solution at 25 °C for 6, 12, 24, and 48 h. The concentration of Ni(II) ions released from Si@NiO increased from 8.01 ppm at 6 h to 18.04 ppm after 48 h (Figure 5). Si@NiO was found to be robust, maintaining its flower-like structure well in 0.9% saline solution.

### 3.6. Anticancer Properties and Cytotoxicity

**Figure 6:**
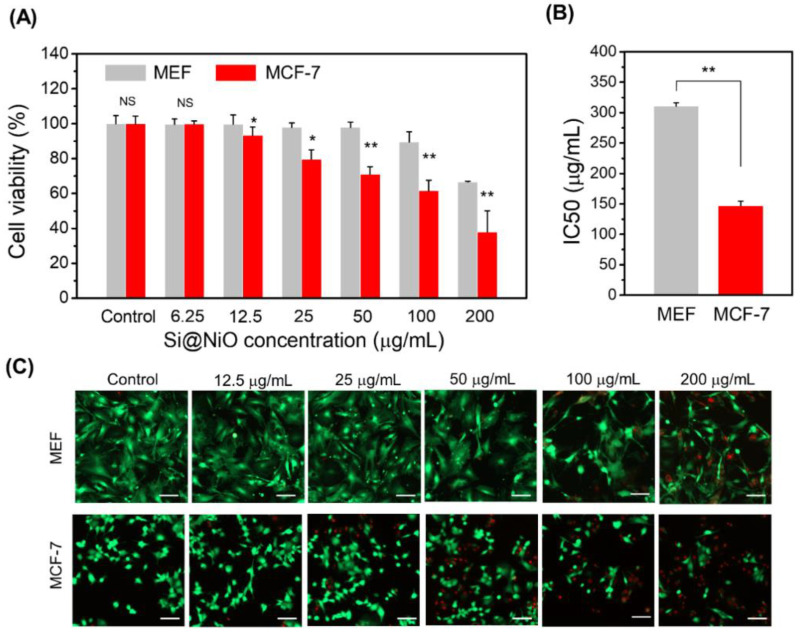
Cytotoxicity and anticancer activity of Si@NiO nanoflowers. (**A**) Cytotoxicity of Si@NiO toward the MEF and MCF-7 cells after 24 h of incubation. (**B**) IC_50_ values of Si@NiO in the MEF and MCF-7 cells. (**C**) Live/dead staining images of MEFs and MCF-7 after incubation with various concentrations of Si@NiO for 24 h. Positive control: cells cultured in the absence of Si@NiO. NS: not significant, * *p* < 0.05 and ** *p* < 0.01. Scale bar: 100 μm.

The most important requirements of cancer therapeutics are efficacy and selective toxicity [58,59]. The ability of a drug to induce cancer cell apoptosis without affecting normal cells is the key requirement for effective cancer treatment. Since the active metal ions on the surface of metal oxide nanoflowers can actively participate in ROS formation through oxidation, these nanoflowers have great potential as a therapeutic for cancer treatment. Moreover, as the metal ion release is not sufficiently high enough, this aspect should not affect the anticancer activity remarkably. Nonetheless, it is important to determine the cytotoxicity of Si@NiO nanoflowers on normal cells and cancer cells. The MCF-7 cancer cell line and MEF normal cell line were used for in vitro anticancer tests [58,60]. The results of the MTS assay are shown in Figure 6A. There were no obvious toxic effects on the MEFs in the presence of 0−100 μg/mL Si@NiO MEFs, and the cell viability was higher than 90% after 24 h of incubation. However, at a higher Si@NiO concentration of 200 μg/mL, the viability of these cells decreased to 67%. At this concentration, the nanoflowers can internalize into the MEF cells and affect them by reacting with the low levels of H_2_O_2_ [60]. Importantly, the viability of the MCF-7 cells decreased and the anticancer activity of Si@NiO against the MCF-7 cells significantly increased depending on the dose of Si@NiO. At the Si@NiO concentrations of 25, 50, 100, and 200 µg/mL, 20%, 29%, 38%, and 62% of cancer cells were killed. The half-maximum inhibitory concentration values (IC_50_) of Si@NiO are shown in Figure 6B; it is evident that Si@NiO nanoflowers are significantly more toxic to MCF-7 cells than they are to MEF cells, with IC_50_ values of 147 and 310 μg/mL for the MCF-7 and MEF cells, respectively. We also performed a live/dead assay to reconfirm the cytotoxicity of the Si@NiO nanoflowers on the normal cells and cancer cells. In the range of 0−100 μg/mL Si@NiO, no obvious toxic effect on the MEFs was observed, and the viability was more than 90% after 24 h of incubation. However, at a higher Si@NiO concentration of 200 μg/mL, the viability of the normal cells decreased to 75%. On the other hand, the viability of the MCF-7 cells decreased depending on the dose of Si@NiO. The MCF-7 cell viabilities at 25, 50, 100, and 200 µg/mL of Si@NiO were 81%, 73%, 49%, and 35%, respectively. These observations agree with the results of the MTS assay. It is likely the ROS that were initially produced during the metabolism of O_2_ by the living systems derived from the superoxide anion. Generally, cancer cells have higher levels of free radicals than normal cells, and this phenomenon is associated with the increased metabolism of cancer cells and mitochondrial dysfunction. Increased free radical production in cancer cells brings about the biochemical and molecular changes that are necessary for cancer cells to become resistant to chemotherapy along with the development, proliferation, and metastasis of cancer cells. Increasing reactive oxygen species to a level that causes cytotoxicity in cancer cells can be an effective method for cancer cell removal by active oxygen-mediated apoptosis or by inhibiting cancer cell resistance [61,62]. Therefore, the ROS formed during the reduction of Ni (II) on the surface of the metal oxide (NiO) are more responsible for the toxic effects on the cancer cells than on the normal cells. Considering the ion release capacity discussed in Section 3.5 and the facile formation of ROS on the surface of the nanoflowers (Section 3.4), it can be speculated that the excellent anticancer activity of Si@NiO originates from its high surface area as well as from the potent oxidative capacity of the metal ions on the surface or that are released from the surface of the Si@NiO nanoflowers rather than from the inflow of metal ions released from Si@NiO into the cells.

## 4. Conclusions

In this work, Si@NiO nanoflowers were prepared as a novel anticancer agent using a modified CBD process followed by thermal reduction. The surface area, redox reaction with AA, metal ion-release properties, and morphology of these nanoflowers were investigated, and reasonable anticancer activity was justified based on these parameters. The CV data suggested that the ROS formation by the oxidants (i.e., Si@NiO nanoflower) might partly contribute to cancer cell killing. The cancer cell killing could primarily be attributed to the direct contact of the nanoflower surface with the cancer cell membranes or to the ROS species formed by the Ni (II) ions on the surface of the nanoflowers. In this respect, both high surface area and the positively charged metals on the surface might be determinant factors in inhibiting cancer cell growth. The considerable anticancer activity toward the MCF-7 cells and the high biocompatibility with the MEF cells suggest their potential applications in cancer therapy and pharmaceuticals. By the virtue of direct contact with cancer cells, further research on nanoflowers containing various metals is expected to broaden the scope of therapeutic technology.

## Figures and Tables

**Table 1:** Micro pore volume (V_micro_), mesopore volume (V_meso_), specific surface area (S_BET_), and average pore size of Si and Si@NiO.

Sample	V_micro_ (cm^3^/g)	V_meso_ (cm^3^/g)	S_BET_ (m^2^/g)	Average pore size (nm)
Si NP	0.006	0.05	23.50	0.56
Si@NiO	0.01	0.59	206.08	6.51

## Data Availability

Data of the study are available from the corresponding author upon reasonable request.
